# Staying well after depression: trial design and protocol

**DOI:** 10.1186/1471-244X-10-23

**Published:** 2010-03-19

**Authors:** J Mark G Williams, Ian T Russell, Catherine Crane, Daphne Russell, Chris J Whitaker, Danielle S Duggan, Thorsten Barnhofer, Melanie JV Fennell, Rebecca Crane, Sarah Silverton

**Affiliations:** 1 Department of Psychiatry, University of Oxford, Warneford Hospital, Oxford, OX3 7JX, UK; 2 West Wales Organisation for Rigorous Trials in Health, School of Medicine, Swansea University, Swansea, SA2 8PP, UK; 3 North Wales Organisation for Randomised Trials in Health, Bangor University, Bangor, Gwynedd, LL57 2HX, UK; 4 School of Psychology, Bangor University, Gwynedd, LL57 1UT, UK

## Abstract

**Background:**

Depression is often a chronic relapsing condition, with relapse rates of 50-80% in those who have been depressed before. This is particularly problematic for those who become suicidal when depressed since habitual recurrence of suicidal thoughts increases likelihood of further acute suicidal episodes. Therefore the question how to prevent relapse is of particular urgency in this group.

**Methods/Design:**

This trial compares Mindfulness-Based Cognitive Therapy (MBCT), a novel form of treatment combining mindfulness meditation and cognitive therapy for depression, with both Cognitive Psycho-Education (CPE), an equally plausible cognitive treatment but without meditation, and treatment as usual (TAU). It will test whether MBCT reduces the risk of relapse in recurrently depressed patients and the incidence of suicidal symptoms in those with a history of suicidality who do relapse. It recruits participants, screens them by telephone for main inclusion and exclusion criteria and, if they are eligible, invites them to a pre-treatment session to assess eligibility in more detail. This trial allocates eligible participants at random between MBCT and TAU, CPE and TAU, and TAU alone in a ratio of 2:2:1, stratified by presence of suicidal ideation or behaviour and current anti-depressant use. We aim to recruit sufficient participants to allow for retention of 300 following attrition. We deliver both active treatments in groups meeting for two hours every week for eight weeks. We shall estimate effects on rates of relapse and suicidal symptoms over 12 months following treatment and assess clinical status immediately after treatment, and three, six, nine and twelve months thereafter.

**Discussion:**

This will be the first trial of MBCT to investigate whether MCBT is effective in preventing relapse to depression when compared with a control psychological treatment of equal plausibility; and to explore the use of MBCT for the most severe recurrent depression - that in people who become suicidal when depressed.

**Trial Registration:**

Current Controlled Trials: ISRCTN97185214.

## Background

Suicidal behaviour is a serious outcome of psychiatric illness in general, and is specifically associated with depression. It has been shown that the population attributable ratio (PAR) for depression in suicidal behaviour is 80 per cent (i.e. 80% of suicidal behaviour would be removed if depression did not occur [1]), and one in seven patients admitted to hospital for major depression will go on to die by suicide. Suicide ideation is one of the most consistently recurring symptoms of depression [2] and combined with the high risk of recurrence of depression (rising to 90% in people with 3 or more previous episodes), makes treatment of patients who have experienced suicidal depression extremely important.

Treatments designed to target suicidal behaviour have had mixed results [3]. Preventing recurrence of suicidal depression is likely to depend on being able to target the factors that underlie continuing vulnerability. Depression is one such vulnerability factor as most suicidal behaviour occurs in the context of depressed mood. However, it would also be useful to target specific vulnerability factors for suicidality directly.

Mindfulness Based Cognitive Therapy (MBCT, [4]) is a manualised treatment programme which has been shown to prevent relapse to depression in those who have experienced three or more depressive episodes in the past [5,6]), and is now recommended by the UK government National Institute for Health and Clinical Excellence as a treatment for prevention of depression recurrence in those with three or more previous episodes. However no studies to date have explored whether MBCT is effective in preventing relapse to depression when compared with a control psychological treatment of equal plausibility. Further the use of MBCT for the most severe recurrent depression - those people who become suicidal when depressed - has not been considered separately in previous published RCTs because participants have not been stratified according to history of suicidal ideation or behaviour.

Recently Williams and colleagues (Fennell, Barnhofer, R. Crane and Silverton) adapted MBCT for use with patients suffering from recurrent suicidal depression (suicidal ideation or behaviour), to address this problem more directly. This treatment showed positive results in a pilot trial by significantly changing mood [7] and measures of ongoing psychological vulnerability from before treatment to after [8,9]. However, this previous trial did not include a follow-up, so we do not yet know if the change in vulnerability led to a reduction in relapse to depression and suicidality.

## Trial Objectives and Purpose

The objectives of this trial are to test whether Mindfulness-Based Cognitive Therapy (MBCT), a novel form of treatment combining mindfulness meditation and cognitive therapy for depression delivered in addition to treatment as usual (TAU), will reduce the risk of relapse to Major Depression in recurrently depressed patients and the incidence of suicidal symptoms in those with a history of suicidality who relapse. To this end we aim to compare MBCT with both TAU alone and Cognitive Psycho-Education (CPE), an equally plausible cognitive treatment, but without meditation.

Thus the study uses a "dismantling" paradigm in the sense that it removes meditation - the component of MBCT, the main treatment under investigation, that we hypothesize to be effective - to form CPE, the comparison treatment, which is otherwise identical in its content. Removal of the meditation component of MBCT from CPE allows us to assess, for the first time, the added benefits of the meditation component of MBCT in preventing relapse in people with a history of recurrent depression.

The study is also investigating potential mediators and moderators of treatment outcome by assessing symptoms, stressful life events, and aspects of cognitive functioning related to risk of relapse to depression - before and after treatment and during the follow-up period.

## Methods/Design

This is a multi-centre, randomised controlled trial. We randomise participants between Mindfulness-Based Cognitive Therapy (MBCT) in addition to Treatment As Usual (TAU), Cognitive Psycho-Education (CPE) in addition to TAU, and TAU alone. In doing so, we stratify participants by (a) research centre (Oxford or Bangor) (b) cohort (c) history of suicidality (none, ideation or suicidal attempt) and (d) whether or not they were taking antidepressants in the 7 days before their first assessment. We originally proposed to stratify by centre, cohort, suicidal behaviour (ideation versus attempt), number of previous episodes of depression (3 or 4 versus 5 or more), and brooding (high versus low scores on a subscale of the Ruminative Response Style questionnaire). However we later changed these, with ethical approval, to reflect the inclusion of participants taking antidepressants or without suicidal ideation. We undertake randomisation by e-mail to the remote randomisation centre at the North Wales Organisation for Randomised Trials in Health (NWORTH) at Bangor University. The randomisation algorithm uses dynamic allocation to protect against subversion while ensuring that each arm of the trial is comparable with respect to the stratification variables. For validation the randomisation e-mail also includes additional information including the participant's date of birth, gender and date of assessment.

**Figure 1:**
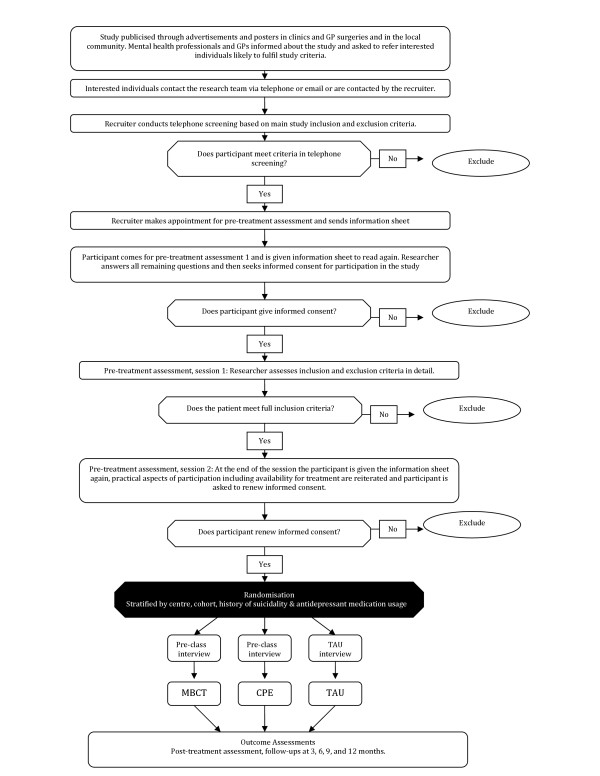
**Diagram showing participant flow through the trial**. This file includes a diagram which outlines participant flow through the trial, from initial contact with the research team to completion of follow-up assessments.

We monitor treatment effects over a period of one year. Assessments by trained assessors who are blind to treatment allocation (and whose blindness is checked within each assessment session) take place directly before and after the start of the treatment (T0 and T1), and at three (T2), six (T3), nine (T4) and twelve months (T5) after T1 (see Figure 1 for details of participant flow through the trial). We also offer treatment 'reunions' to participants in both MBCT and CPE at both 6-8 weeks and 6 months after treatment.

The study is being conducted in accordance with the WMA Declaration of Helsinki and has been approved by the National Research Ethics Service (Oxfordshire REC C) (MREC 08/H0606/56) and the North Wales Research Ethics Committee.

### The Interventions

The two treatments, MBCT and CPE, both consist of 8 weekly classes of two hours duration. MBCT [4] is a manualised treatment programme that combines training in mindfulness meditation with cognitive therapy techniques which has been adapted for this trial by Williams and colleagues (Melanie Fennell, Rebecca Crane, Thorsten Barnhofer and Sarah Silverton) for patients with a history of suicidality. In addition to weekly meetings, we advise participants to spend about an hour per day on home-based practice which includes regular meditation practice and smaller tasks aimed at cultivating mindfulness in everyday life. The rationale of the treatment is based on research suggesting that, in vulnerable individuals, negative thinking patterns can be easily re-activated through only minor events like subtle changes in mood, and that negative mood is often perpetuated through maladaptive habitual patterns of thinking that are characterized by ruminative tendencies and avoidance. Through meditation practice, MBCT aims to help participants to become aware of re-activation of negative thinking at earlier stages and to disengage from maladaptive reactions such as ruminative and avoidant tendencies, which might otherwise ensue. Participants learn to relate to all their experience with openness and acceptance. MBCT has been found to reduce successfully the risk of relapse in patients with three or more episodes of depression in the past. Previous work from our group has shown that cognitive reactivity is particularly pronounced in patients who have suffered from suicidal depression in the past [10], suggesting that MBCT might be particularly helpful as a treatment approach to reduce vulnerability in this group.

The CPE program uses the same format of eight weekly group meetings of two hours. It includes all the elements of the MBCT programme *except *those that are intended to support participants in experientially cultivating mindfulness, namely the meditation practices and the focus in the sessions on experiencing in the present moment. In CPE, as in MBCT, participants are taught about the psychological processes in relapse to depression, and engage in mood monitoring and homework to prevent relapse. They are also taught about the importance of recognising the various elements of experience (thoughts, emotions, sensations and behaviours) and of disengaging from unhelpful patterns of processing such as rumination and experiential avoidance. However, instead of training different ways of relating to these experiences through meditation, CPE *informs *participants about these processes through teacher-led presentations and group discussions. In summary, the main themes of the MBCT and CPE sessions centre on the same subjects and cover the same points; CPE differs from MBCT in avoiding meditation training in the sessions or through home practice. In addition to allowing 'dismantling' analyses of the role of meditation in MBCT, investigating CPE is important as it constitutes an economical alternative treatment.

The four therapists deliver both MBCT and CPE, alternating between the two treatments across the 6 classes that each leads. In week 2 of treatment we ask participants to rate the plausibility of their treatment, not only to assess whether the two treatments are equally plausible, but also to check for therapist treatment allegiance effects. Additionally treatment adherence and competence are being monitored by JMG Williams, who developed MBCT [4].

### Sample Size

Two previous trials investigating MBCT for those people who have experienced 3 or more episodes of depression in the past have found that MBCT reduces recurrence from 70% to 39% compared with TAU [5,6]. In this trial we expect that the difference between MBCT and CPE, the other primary comparison, will be smaller. Bockting and colleagues [11] found that Cognitive Behaviour Therapy delivered in groups reduced relapse from 65% (TAU) to 54%; using the hazard ratio derived from these figures, we would expect CPE to reduce rates of relapse or recurrence from 70% to 59%. This difference in effectiveness between CPE and TAU led us to allocate patients between MBCT, CPE and TAU in the ratio 2:2:1. Using a 5% significance level, a final sample of 300 participants (120 MBCT, 120 CPE and 60 TAU) yields 99% power for survival analysis to detect a difference in recurrence between 70% in the TAU control group and 39% in the MBCT group, and 80% power to detect a difference between 39% in the MBCT group and 57% in the CPE group - less than the 59% suggested above.

Although participants are individually randomised, MBCT and CPE, but not TAU, are administered in groups, which means that participants within the same group may influence each other and observations of outcome within groups may not be independent. Reanalysing the two previous trials [5,6], we found that intra-class correlations (ICCs) for recurrence were less than zero, and therefore have little or no effect on power calculations [12]. For depression measures, ICC varied from zero to less than 0.05. MBCT and CPE participants will form 12 therapy groups (clusters) of each type. For secondary analyses on measured outcomes, with 10 respondents per cluster, the proposed sample sizes give 80% power to detect a difference between MBCT and CPE of 0.45 standard deviations if the ICC is 0.05. The smaller sample available for comparing CPE or MBCT with TAU means that the corresponding detectable effect size is 0.53 if the ICC is 0.05. These are generally considered moderate effect sizes. Given a likely attrition rate of 20%, we shall recruit 375 participants to achieve the final sample of 300.

### Referral and recruitment

We recruit participants through advertisements in the community, in clinics and GP surgeries, as well as through referrals from GPs and mental health clinicians, whom we inform about the study through letters and talks at professional meetings. Recruiters working on the trial contact people who respond to advertisements, GPs or mental health clinicians and explain the study to them. If they express interest, a recruiter screens them for the main inclusion and exclusion criteria of the trial using a standardised checklist. The recruiter sends the information sheet to eligible participants, and, if they are willing to participate, invites them to an assessment session at the trial site in Oxford or Bangor.

### Inclusion criteria

Principal inclusion criteria for the study are:

1) Age between 18 and 70 years, because depression in old age is related to different factors than depression in earlier stages of life [13,14].

2) Meeting enhanced DSM-IV criteria for a history of Recurrent Major Depression, namely a history of at least three episodes of depression, of which two must have occurred within the last five years, and one within the last two years [15]. Although previous suicidality is recorded in detail, thus facilitating stratification, prior experience of suicidality is not a prerequisite for participating in the trial.

3) Meeting the NIMH guidelines for recovery or remission at the time of baseline assessment. Potential trial participants are deemed *not *to be in recovery or remission, and hence *ineligible*, if they report at least one week during the previous 8 during which they experienced *either *a core symptom of depression (depressed mood, anhedonia) *or *suicidal feelings and at least one other symptom of depression, which together are not attributable to bereavement, substances or medical condition, but are impairing functioning.

4) Giving informed consent.

5) Consent received from the participant's General Practitioner.

### Exclusion criteria

We exclude potential trial participants if one or more of the following apply:

1) They have a history of schizophrenia, schizoaffective disorder, bipolar I disorder, current severe substance abuse, organic mental disorder, pervasive developmental delay, a primary diagnosis of obsessive-compulsive disorder or eating disorder, or regularly harm themselves.

2) They report a positive continuing response to CBT.

3) They receive psychotherapy or counselling more than once per month

4) They cannot complete the baseline research assessment, for example through difficulties with English, visual impairment, or cognitive difficulties.

### Assessment of Eligibility and Baseline Measures

Before the first assessment session, researchers explain the trial to participants and give them the information sheet again. After they have had opportunity to discuss any questions, the researchers seek written informed consent after telling participants that they have the right to withdraw from the research at any time.

We ask consenting participants to provide information about their socio-demographic background and assess their eligibility in more detail using semi-structured clinical interviews and self-completed questionnaires. The researchers assess current and past diagnostic status using the Structured Clinical Interview for DSM IV (SCID [16]), the Suicide Attempt Self Injury Interview (SASII [17]) and the Hamilton Rating Scale for Depression (HRSD[18]). They ask participants to complete a crisis card, and to describe past and current treatments for depression and past meditation and yoga experience.

They then ask participants themselves to complete the Beck Scale for Suicide Ideation (BSS-current [19] and worst [20]), the Mini International Neuropsychiatric Interview (MINI) Suicidality Tracking measure [21], the Beck Depression Inventory (BDI-II [22]) to assess severity of current symptoms of depression, the Beck Hopelessness Scale (BHS [23]) to measure current levels of hopelessness, a questionnaire assessing occurrence of life events [24], a measure of global functioning (Clinical Outcome Routine Evaluation, CORE [25]), a measure of general quality of life (Euro-QOL EQ-5D [26]), a questionnaire assessing history of trauma (Childhood Trauma Questionnaire, CTQ [27]), and two short measures of anxiety and depression symptoms (GAD7 [28] and Patient Health Questionnaire, 9-item version, PHQ9 [29]). If participants meet all inclusion and none of the exclusion criteria for the study, they enter the study and are invited to the second pre-treatment assessment session.

The second pre-treatment assessment asks participants to complete several cognitive tasks and to fill in several self-completed measures assessing factors related to cognitive vulnerability for depression. The cognitive tasks include the Autobiographical Memory Test (AMT [30]), in which participants are given cue words and asked to remember specific events from their life. They also complete two short tests of executive capacity, the Number Generation Task [31], in which they generate sequences of numbers within given ranges, and the Baddeley Dual Task [32]. Questionnaires at this assessment include measures of: mindfulness (Five Factor Mindfulness Questionnaire, FFMQ [33]), self-compassion (Self-Compassion Scale, CS [34]), rumination (Ruminative Responses Subscale of the Response Styles Questionnaire, RSQ [35]), dysfunctional attitudes (Dysfunctional Attitudes Scale, DAS [36]), acceptance (Acceptance and Action Questionnaire, AAQ [37]), suicidal cognitions (Suicide Cognitions Scale, SCS, 37), suicidal thinking (Suicidal Thoughts Questionnaire), self-discrepancies (Self-Guides Questionnaire [38]) and frequency of thought suppression.

### Informed Consent

We seek informed consent on two occasions, the first before assessing eligibility, already described. Secondly, we ask participants who are eligible and have finished the second pre-treatment assessment to renew their consent *before *they are randomised to one of the treatments. Participants again receive full information about the study and the opportunity to ask any questions about the trial. We remind them that they can withdraw from the trial at any time without affecting their usual care. The researchers check that participants understand all aspects of the trial. If they agree to enter the trial, they complete another three copies of the consent form. One copy of the completed consent form is for the participant, one for the local research team, and the last for the central research team in Oxford.

### Outcome Measures

The primary outcome measure of the trial is the time to relapse or recurrence meeting DSM-IV criteria for Major Depression, which we assess by the Structured Clinical Interview for DSM-IV (SCID). We assess the occurrence of relapse or recurrence at all follow-up assessments, and treat 'return to treatment' as a relapse or recurrence if, in the judgment of a blind assessor, the participant has experienced exacerbation of symptoms that would have met the criteria for Major Depression in the absence of immediate treatment. In addition to diagnostic status, we assess severity of depression and hopelessness at all time points, using several interview and self-completed measures including the Hamilton Rating Scale for Depression, the Beck Depression Inventory and the Beck Hopelessness Scale. These quantitative measures strengthen the dichotomised outcome of diagnosis. The statistical analysis plan in Appendix 1 gives further details.

We assess cognitive measures relevant to risk of relapse or recurrence, namely mindfulness, self-compassion, rumination, self discrepancy, autobiographical memory and executive capacity before and immediately after treatment and at the end of the follow-up. We shall use these measures in explanatory analysis of factors that mediate or moderate efficacy.

### Pre-Class Interviews

We tell all participants the outcome of randomisation by letter (in addition to email or telephone if requested). We invite those allocated to MBCT or CPE to meet the therapist running their class and those in TAU to meet another member of the research team. These meetings take between 1 and 1.5 hours and either prepare participants for classes, including discussion of ways of coping with barriers to treatment, or discuss procedures for keeping in touch with participants not in classes.

Each week of the eight-week treatment phase, we ask all participants to complete a brief set of tracking questionnaires. These include short ratings of tendencies to ruminate, occurrence of intrusive thoughts, thought suppression, and the PHQ9 and GAD7 scales for assessing symptoms of depression and anxiety. At the start of the second class we also ask participants receiving MBCT or CPE to rate the plausibility of their treatment. Finally participants receiving MBCT complete a short diary documenting homework and practice completed throughout the week.

### Post-Treatment Assessment and Follow-Ups

Once treatment is finished, we invite all participants to a post-treatment assessment. Over the following 12 months we invite them to attend research assessments at three, six, nine and twelve months after treatment. We also invite those allocated to treatment with MBCT or CPE to attend two treatment sessions, one 6-8 weeks, the other six months after treatment. At post-treatment assessment (T1), we use the SCID mood disorder module to assess current diagnostic status and any changes in that status over the treatment phase. We ask participants to repeat the cognitive tasks and questionnaires they completed during the pre-treatment assessments with the exception of the Childhood Trauma Questionnaire (CTQ), the worst ever BSS, the crisis card, and questions about sociodemographic status. The follow up assessments at three (T2), six (T3) and nine months (T4) consist of a SCID mood disorder module, the Hamilton Rating Scale for Depression and, when there has been suicidal behaviour or self harm, the SASII assessment, focusing on the time since the last assessment. We also ask them to complete questionnaires assessing current symptoms and history of treatment since last assessment (BSS current, Suicide Cognitions Questionnaire, MINI Suicidality Tracking, BDI-II, BHS, Life Events Questionnaire, CORE, Euro-QOL, PHQ9, GAD7). During the final follow-up assessment (T5), we ask participants to complete the same measures as at the T1 assessment. Additional File 1 includes a table summarizing all these baseline and follow-up assessments.

### Withdrawal

Participants can withdraw from treatment or data collection or both at any time without having to give a reason. Nevertheless we ask those who withdraw from the trial treatment (MBCT or CPE) to attend all the remaining research appointments or at least to provide minimal data if they are willing.

### Safety monitoring and reporting

We record and report suspected serious adverse events to the Trial Steering Committee (TSC), the Data Monitoring and Ethics Committee (DMEC), and serious adverse reactions to the Multi-centre Research Ethic Committee according to their individual guidelines.

### Analysis

We shall analyse all data by intention to treat. We shall use Cox regression, a form of survival analysis that takes account of covariates, to analyse relapse and recurrence. We shall analyse most other measured variables by mixed-model analysis of variance (ANOVA). We shall use baseline values of the dependent variable as a covariate in all analyses; we shall use other baseline measures or demographic characteristics as covariates when they contribute significantly to the analysis. We shall use multi-level modeling to take account of the clustering of participants within classes within centres. To minimise testing, we shall combine measured outcomes at different time points using the 'area under (the resulting) curve'. We shall use cognitive measures to explore the extent to which they mediate relapse and recurrence during treatment and follow up. Similarly we shall use data at each time-point on the Beck Scale for Suicide Ideation to explore the extent to which the cognitive measures mediate the occurrence of suicidality over the follow-up period, again analysing 'area under the curve'. Additionally we shall examine recurrence of suicidality specifically for those participants who relapse to Major Depression (MDD) and had a history of suicidal ideation or behaviour at entry to the trial. We predict that, all else being equal, suicidal ideation will fall in those who have received treatment with MBCT. Further details of the data analysis plans are given in Appendix 1.

## Discussion

Recurrent depression is highly prevalent and reducing risk of relapse is of particular importance in those who are likely to become suicidal when depressed. This trial will be the first to evaluate the efficacy of Mindfulness-based Cognitive Therapy (MBCT) for this population, and the first to use a design that compares MBCT with both an active 'control' treatment and usual care. This trial will provide the opportunity to investigate further treatment approaches known to be promising and to learn more about mechanisms of treatment in order to refine this approach. The use of a 'dismantling design' for this purpose is unusual.

We selected the 'control' treatment from several options. One was to compare the MBCT 'package' with an alternative group-based package, for example group-based CBT that would entail comparable group attendance and homework assignments. There are two reasons why we rejected this option. First, treatments like CBT were designed to treat acute depression rather than prevent relapse, and appear to be less effective in preventing relapse [11]). Secondly, whatever the outcome of such a comparison, we could not answer our key scientific question: which component of relapse-prevention treatment is critical to success?

In a 'dismantling' paradigm, the comparison treatment is identical to the index treatment, but has a critical component removed. Given that the most complex aspect of MBCT is intensive training in meditation, we decided that the control treatment should follow the same group format as MBCT but *without *any training in meditation. Thus participants have the same number and length of sessions as MBCT, controlling for group and therapist support, but with short lecture-type presentations and group discussions instead of meditation training. These cover the psycho-educational components of learning about depression, links between thoughts and feelings, and how to self-monitor these for signs of impending recurrence. We have developed a rigorous manual for this treatment package and piloted it in both Oxford and Bangor. Note that a dismantling design does not match treatments for homework assignments since meditation needs more homework. If MBCT is more effective than CPE, then it will be for further research to address the question whether homework would have enhanced efficacy.

The intention in this trial is to examine MBCT for people with severe recurrent depression, including suicidal ideation or behaviour. We considered including only participants who at the outset acknowledge experience of suicide ideation or behaviour. However the stigma associated with suicidality and the frequent failure to disclose suicidal thoughts and behaviours to clinical staff, made us suspicious that this would reduce recruitment to the trial. For example consultation with GP practices revealed reluctance to circulate information about a trial explicitly targeting suicidal depression to patients whose practice notes might not record such a history. We were also concerned that people would be less likely to refer themselves to the trial if posters and other material focused on suicidal ideation or behaviour. Although the stigma surrounding suicidal ideation and behaviour is unfortunate and should be challenged, recruitment is crucial in trials. So, although the special interest in suicidal depression is shared with referring clinicians, recruitment is open to all who have experienced recurrent depression, rather than specifying minimum levels of suicidality as an inclusion criterion. We then stratify participants according to history of suicidality reported on standardised measures used in the initial assessments of eligibility, and use these measures as covariates in the definitive analysis. We still expect most participants to report some history of suicide ideation or behaviour, even though this is not explicit in recruitment materials.

We encourage all participants to continue their 'treatment as usual' as determined through consultations with their GP and other mental health professionals, for the duration of the study. In addition we ask those not allocated to MBCT not to take up a regular meditation practice over the year of follow-up, and thus to restrict their choice of treatment. As we cannot enforce this in practice, we ask at each assessment whether participants have taken up any treatment or meditation practices so that we can take this into account in analysis. Furthermore we shall offer all participants, especially those receiving TAU alone, treatment classes of their choice after the end of the study. The research team will maintain contact with those in the TAU group throughout the trial, and encourage them to make full use of the services available to them. As participants may relapse to suicidal depression during the study, we explain the limits of confidentiality and the procedure for dealing with severe suicidal ideation to all participants before asking them to give consent.

In summary, recurrent depression is common and gives rise to increased risk of morbidity and mortality for those who become suicidal when depressed. There is an urgent need: first to develop treatments that can address the needs of this group and produce sustainable reductions in risk of recurrence; and, second to identify the critical therapeutic factors in order to refine the approach for the future. This trial will address both objectives.

## Competing interests

The authors declare that they have no competing interests.

## Authors' contributions

JMGW, CC and DD drafted this paper which was added to and modified by all other authors. JMGW and MJVF modified MBCT for suicidal participants and JMGW, MJVF, TB, RC and SS wrote the content of the Cognitive Psycho-Education treatment. JMGW, DR and ITR contributed to the design of the study and CJW to the analytic strategy. All authors read and approved the final manuscript.

## Appendix 1: Statistical Analysis Plan

The primary outcome measure will be the time to relapse or recurrence meeting DSM-IV criteria for a major depressive episode (American Psychiatric Association, 1994) on the Structured Clinical Interview for DSM-IV (SCID, Spitzer et al., 1992). Occurrence of relapse or recurrence will be assessed after treatment (T1), and at three (T2), six (T3), nine (T4) and twelve (T5) months thereafter by trained psychologists blind to participants' treatment condition. If the interview establishes that symptoms meeting diagnostic criteria for major depression have been present since the last assessment, we shall ask participants when this episode of depression started (and ended, if they are no longer symptomatic). 'Return to treatment' will also be treated as a relapse or recurrence if, in the judgment of a blind rater, the participant experienced exacerbation of their symptoms that would have met the criteria for Major Depression in the absence of immediate treatment.

The analysis will be by 'intention to treat' (ITT). The time (in weeks) of relapse or recurrence to Major Depression, as defined above, will be the dependent variable in survival analysis. The treatment group and stratification variables will be used as predictors.

For participants who are lost from the trial we shall use their available measures and then censor them at the time of their last observation. Since only a participant's first relapse or recurrence to Major Depression will contribute to the survival analysis, the subsequent loss of that participant will not affect the analysis.

Participants who miss one or more follow-up assessments, but are then assessed at a later time point will be asked whether they have experienced a relapse or recurrence according to SCID diagnostic criteria since the last assessment, including time periods which would have been covered in missed assessments. This will enable us to assess the time to relapse and thus to censoring.

We shall use the clinician-rated Hamilton Rating Scale for Depression (HRSD, to assess severity of depression at all time points. We shall follow De Rubeis et al. (2005) and Hollon et al (2005) in using a score of 14 or more on the HRSD to indicate relapse, thus complementing the SCID diagnosis. HRSD scores also provide a quantitative measure of outcome that strengthens the dichotomised outcome of diagnosis, because, as a quasi-continuous measure, it has more power to detect differences between groups.

The other quantitative measures used at baseline, before treatment, and at times T1 to T5 are the Beck Depression Inventory (BDI-II), Beck Hopelessness Scale (BHS), Beck Scale for Suicide Ideation (BSS current) and the EQ5D. We shall calculate the 'area under the curve' (AUC) of each measure to give a single score. For the EQ5D this is known as a QALY.

Missing items within individual outcome measures will be treated according to the instructions for that measure. If two or more observations for quantitative measures are available between T1 and T5, then we shall use linear regression to estimate the missing values and the AUC. If there is only one observation available between T1 and T5 then we shall estimate the remaining values from the general slope estimated from all participants with two or more observations. If no observations for T1 to T5 are available then the AUC is also missing. If the trend among participants with full data departs from linear, then we shall use the observed trend to estimate the missing values.

For the quantitative measures we shall use a mixed-model analysis of covariance (AnCova). We shall use baseline measures as covariates, and multi-level modeling to take account of the clustering of participants within classes within centres. As covariates we shall also use the stratification variables and treatment group, together with the number of sessions attended and, in the MBCT group, number of hours of home practice. These will allow us to estimate how the response to the two treatments depends on their 'dose'.

Potential moderators to be examined include gender, residual symptoms of depression (e.g. HRSD score and BDI-II score at baseline) and stability of remission, course of previous history (chronic vs episodic), and age of onset.

Recurrence of suicidal ideation, both within episodes of major depression and over the follow-up period, is an important secondary outcome. Initially we shall examine recurrence of suicidality specifically for those participants who relapse to Major Depression and had a history of suicidal ideation or behaviour at entry to the trial. Then we shall compare severity of suicidal symptoms, as measured by the Beck Scale for Suicide Ideation (BSS current) and the MINI suicide-tracking measure, across the follow-up period for all participants, whether or not they relapsed or became suicidal. Finally we shall compare suicidal cognitions (and ability to let go of cognitions that occur), at baseline, T1 and T5 only between groups using AnCova, with T0 as covariate and T1 and T5 as separate outcomes.

By assessing cognitive measures relevant to risk of relapse to depression, namely mindfulness, suppression, self-compassion, rumination, self guides, autobiographical memory and executive capacity, before and immediately after treatment and at the end of follow-up, we can use them in an explanatory analysis to study factors that mediate efficacy. We shall use regression analysis (binary logistic regression for the dichotomous outcome of relapse and linear regression for the worst HRSD score during follow-up) to explore whether the change from T0 to T1 in each of these measures can account for the effects of treatment on risk of relapse.

Where necessary we shall transform variables closer to Normal distributions for AnCova. Where data are missing, we shall use data from alternative sources, notably therapists on the trial and referring general practitioners. We shall also use sensitivity analysis to assess the effect of including participants whose data on relapse is collected by these means.

## Pre-publication history

The pre-publication history for this paper can be accessed here:

http://www.biomedcentral.com/1471-244X/10/23/prepub

## Supplementary Material

Additional file 1 **Measures used at each trial assessment**. This file includes a table outlining the measures completed by participants at each trial assessment. Click here for file
