# Supplementary material for: Staying well after depression: trial design and protocol
Source: BMC Psychiatry. 2010 Mar 19;10:23. doi: 10.1186/1471-244X-10-23 (PMC2859374; doi:10.1186/1471-244X-10-23)
Supplement: Additional file 1 — Measures used at each trial assessment. This file includes a table outlining the measures completed by participants at each trial assessment. [file 1471-244X-10-23-S1.DOC]

**Table 1:** Overview of trial assessments.

| **Topic** | **Measure** | **Type** | **T0** | **Tr** | **T1** | **T2** | **T3** | **T4** | **T5** |
| --- | --- | --- | --- | --- | --- | --- | --- | --- | --- |
| *Clinical Status* | |  |  |  |  |  |  |  |  |
| Socio-demographic Questions | | Int | x |  |  |  |  |  |  |
| Structured Clinical Interview for DSM-IV (SCID) | | Int | x |  |  |  |  |  |  |
| SCID module A (Mood Disorders) | | Int |  |  | x | x | x | x | x |
| Questions on previous or recent treatment, including questions on meditation/yoga experience | | Int | x |  | x | x | x | x | x |
| Hamilton Rating Scale for Depression (HRSD) | | Int | x |  | x | x | x | x | x |
| Suicide Attempt Self Injury Interview (SASII) & MINI suicidality tracking | | Int | x |  | x | x | x | x | x |
| Crisis Card | | Int | x |  |  |  |  |  |  |
| Beck Scale for Suicide Ideation – Current (BSS Current) | | Int | x |  | x | x | x | x | x |
| Beck Depression Inventory (BDI) | | Quest | x |  | x | x | x | x | x |
| Beck Hopelessness Scale (BHS) | | Quest | x |  | x | x | x | x | x |
| Life Events Questionnaire | | Quest | x |  | x | x | x | x | x |
| Clinical Outcome Routine Evaluation (CORE) | | Quest | x |  | x | x | x | x | x |
| Quality of Life Questionnaire (Euro-QOL-5 item) | | Quest | x |  | x | x | x | x | x |
| Beck Scale for Suicide Ideation – Worst Ever (BSS Worst Ever) | | Quest | x |  |  |  |  |  |  |
| Childhood Trauma Questionnaire (CTQ) | | Quest | x |  |  |  |  |  |  |
| PHQ9 | | Quest | x | X | x | x | x | x | x |
| GAD7 | | Quest | x | X | x | x | x | x | x |
| *Vulnerability Factors* | |  |  |  |  |  |  |  |  |
| Five Factor Mindfulness Questionnaire (FFMQ) | | Quest | x |  | x |  |  |  | x |
| Self Compassion Scale (CS) | | Quest | x |  | x |  |  |  | x |
| Self-Discrepancies Questionnaire | | Quest | x |  | x |  |  |  | x |
| Ruminative Response Scale of the Response Style Questionnaire (RRS) | | Quest | x |  | x |  |  |  | x |
| Suicide Cognitions Scale (SCS) & additional questions for endorsed items. | | Quest | x |  | x |  |  |  | x |
| Suicidal Thoughts Questionnaire | | Quest | x |  | x |  |  |  | x |
| Dysfunctional Attitudes Scale (DAS) | | Quest | x |  | x |  |  |  | x |
| Acceptance and Action Questionnaire (AAQ) | | Quest | x |  | x |  |  |  | x |
| Rumination Rating | | Quest |  | X |  |  |  |  |  |
| Rating of Intrusive Thoughts | | Quest |  | X |  |  |  |  |  |
| Suppression of Suicidal Thoughts | | Quest |  | X |  |  |  |  |  |
| *Cognitive Factors* | |  |  |  |  |  |  |  |  |
| Autobiographical Memory Test (AMT) | | Task | x |  | x |  |  |  | x |
| Number Generation Task | | Task | x |  | x |  |  |  | x |
| Baddeley Dual Task | | Task | x |  | x |  |  |  | x |
| *Treatment-related Measures* | |  |  |  |  |  |  |  |  |
| Homework and Practice Diary | | Quest |  | x ^1^ |  |  |  |  |  |
| VAS Measure of Treatment Plausibility | | Quest |  | x ^2^ |  |  |  |  |  |

*Table Notes.* Int = Interview, Quest = questionnaire, T0 = Pre-treatment assessment sessions 1 and 2, Tr = tracking measures completed by ALL participants once per week during the period when CPE and MBCT participants are receiving treatment, T1 = Post-treatment assessment, T2 = 3-month follow-up, T3 = 6-month follow-up, T4 = 9-month follow-up, T5 = 12-month follow-up; ^1^ MBCT and CPE groups only; ^2^ only at the end of the first week of treatment (beginning of session 2) for MBCT and CPE groups.
